# Agronomic Trait Analysis and Genetic Mapping of a New Wheat Semidwarf Gene *Rht-SN33d*

**DOI:** 10.3390/ijms24010583

**Published:** 2022-12-29

**Authors:** Chaojie Wang, Lili Zhang, Yongdun Xie, Ahsan Irshad, Huijun Guo, Jiayu Gu, Linshu Zhao, Hongchun Xiong, Shirong Zhao, Chengshe Wang, Luxiang Liu

**Affiliations:** 1 Institute of Crop Sciences, Chinese Academy of Agricultural Sciences, National Engineering Laboratory for Crop Molecular Breeding, National Center of Space Mutagenesis for Crop Improvement, Beijing 100081, China; 2 College of Agronomy, Northwest A&F University, Yangling, Xianyang 712100, China

**Keywords:** common wheat, dwarf mutant, gene mapping, QTL, *Rht-SN33d*

## Abstract

Plant height is a key agronomic trait that is closely to the plant morphology and lodging resistance in wheat. However, at present, the few dwarf genes widely used in wheat breeding have narrowed wheat genetic diversity. In this study, we selected a semi-dwarf wheat mutant *dwarf33* that exhibits decreased plant height with little serious negative impact on other agronomic traits. Genetic analysis and mutant gene mapping indicated that *dwarf33* contains a new recessive semi-dwarf gene *Rht-SN33d*, which was mapped into ~1.3 Mb interval on the 3DL chromosome. The gibberellin metabolism-related gene *TraesCS3D02G542800*, which encodes gibberellin 2-beta-dioxygenase, is considered a potential candidate gene of *Rht-SN33d*. *Rht-SN33d* reduced plant height by approximately 22.4% in mutant *dwarf33*. Further study revealed that shorter stem cell length may be the main factor causing plant height decrease. In addition, the coleoptile length of *dwarf33* was just 9.3% shorter than that of wild-type Shaannong33. These results will help to expand our understanding of new mechanisms of wheat height regulation, and obtain new germplasm for wheat improvement.

## 1. Introduction

Wheat (*Triticum aestivum* L.) is a gramineae crop, and the third most widely cultivated crop in the world, after rice and maize. As such, enhancing wheat yield remains a major target of wheat breeding. Wheat cultivation conditions have been continuously improved, with more fertilizer and irrigation facilities used to achieve higher yields. However, excessive application of fertilizers and irrigation cause excessive elongation of the internodes, resulting in lodging [1]. Beginning in the 1960s, the widespread application of semi-dwarfing genes in commercial wheat cultivars greatly improved wheat lodging resistance and yield [2]. Therefore, wheat height modification and the molecular mechanism underlying wheat height regulation are important for wheat improvement.

To date, more than 20 quantitative trait loci (QTLs), and a few cloned genes related to wheat plant height regulation, have been reported, such as *Rht1*, *Rht2* [3], *Rht3* [4], *Rht4*, *Rht5*, *Rht12*, *Rht13* [5], *Rht24* [6], and *Rht8* [7,8]. *Rht1* and *Rht2* were widely introduced into modern commercial wheat varieties during the first ‘Green Revolution’. Approximately 24.5% of wheat varieties from autumn-sown regions of China have the gene *Rht1*, and the frequency of *Rht2* is as high as 45.5% [9]. *Rht1* and *Rht2* genes were originally identified from Norin 10 [10]. *Rht1* has been mapped to the short arms of wheat chromosome 4B, and *Rht2* is a homoeologous gene of *Rht1* that was mapped to the short arms of chromosome 4D [3]. According to the present study, the functions underlying *Rht1* and *Rht2* in plant height regulation have been well studied. *RhtB1a* and *RhtD1a*, wild-type alleles of *Rht1* and *Rht2*, encode a protein that contains a DELLA domain at the N-terminus. *RhtB1b* and *RhtD1b*, mutant-type alleles of *Rht1* and *Rht2*, contain a mutation which results in a stop codon within the open reading frame, and produces truncated Rht proteins. The wild-type Rht protein represses plant growth, but this repression can be removed by gibberellin (GA)-induced Rht degradation. However, the truncated Rht protein can avoid degradation and exert a continuous inhibitory effect on plant growth [11]. Recent research has revealed new insights into *Rht1* and *Rht2* [12]. Another widely used dwarf gene, *Rht8*, encodes an RNase H-like protein carrying a zinc finger BED-type motif, has a high distribution in the Yellow and Huai River Valleys, and has been mapped to chromosome 2D [6,7,13]. *Rht3* (the *Rht-B1c*, allele of the gene *Rht1*) is a dominant dwarfing gene and results in a reduction of plant height of up to 46% [14,15]. *Rht10* is an allele of *Rht2* [5]. Other dwarf genes, *Rht4*, *Rht5*, *Rht9*, *Rht12*, and *Rht13*, have also been identified and result in varying degrees of dwarfing [5]. Although the above ~20 dwarfing genes have been identified, only *Rht1*, *Rht2*, *Rht8* and *Rht24* have been widely introduced into commercial varieties [6,9]; this has resulted in a narrow genetic diversity of wheat cultivars. On the other hand, the negative effects of these genes have been widely introduced into commercial varieties. For example, *RhtB1b* and *RhtD1b* result in shortened coleoptile length and reduced seedling vigor [16]. Some results have also revealed that excellent water and fertilizer conditions are necessary for higher production from varieties with the *RhtB1b* or *RhtD1b* allele [17]. Therefore, identifying new dwarf wheat germplasm resources is important and necessary for wheat improvement.

In this study, a dwarf wheat mutant, *dwarf33*, was identified in Shannonong33 (SN33), a commercial wheat variety, using a mutant library induced by the chemical mutagen NaN_3_. *dwarf33* exhibited some good agronomic traits. Interestingly, it had no significant differences in grain length and width compared to SN33, and there was no serious negative impact on coleoptile length. Genetic analysis revealed that *dwarf33* contains a recessive dwarf gene, named *Rht-SN33d,* which is limited to a ~1.3 Mb region at the end of the wheat 3DL chromosome. Gene prediction indicated that the GA metabolic gene *TraesCS3D02G542800,* encoding gibberellin 2-beta-dioxygenase, is the key candidate gene. Overall, the identification of new wheat dwarf germplasm resources and genes not only expand genetic diversity but also provide new germplasm for wheat breeding. This study also provides a potential target gene for improving wheat varieties and production using molecular biological techniques.

## 2. Results

### 2.1. dwarf33 Shows Decreased Plant Height

**Figure 1:**
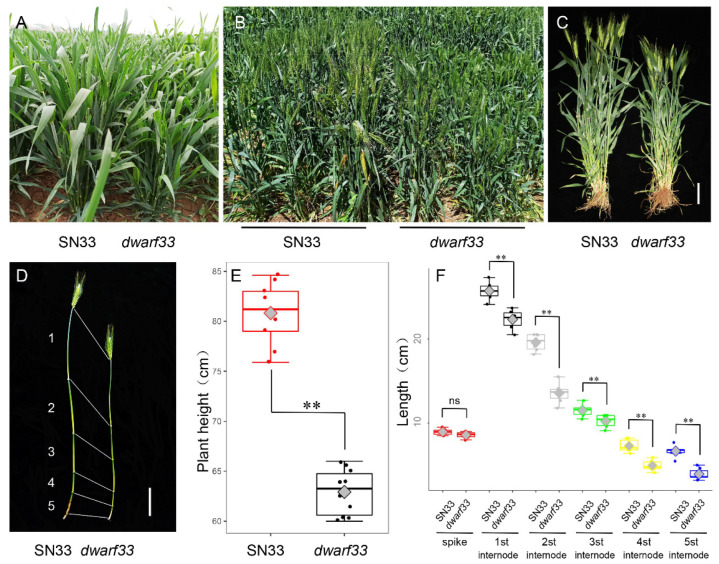
Phenotypes of SN33 and *dwarf33* under field conditions. (**A**) Plant height of SN33 and *dwarf33* at the jointing stage. (**B**) Plant height of SN33 and *dwarf33* at the flowering period. (**C**) Plant height of SN33 and *dwarf33* at the filling stage. (**D**) Internode length of SN33 and *dwarf33* at the filling stage. (**E**) Plant height analysis of SN33 and *dwarf33* at the filling stage. (**F**) Spike and internode length analysis of SN33 and *dwarf33* at the filling stage. Label = 10 cm; “ns” indicates no significant differences between SN33 and *dwarf33*; Asterisks “**” indicate significant differences at *p* ≤ 0.01.

**Figure 2:**
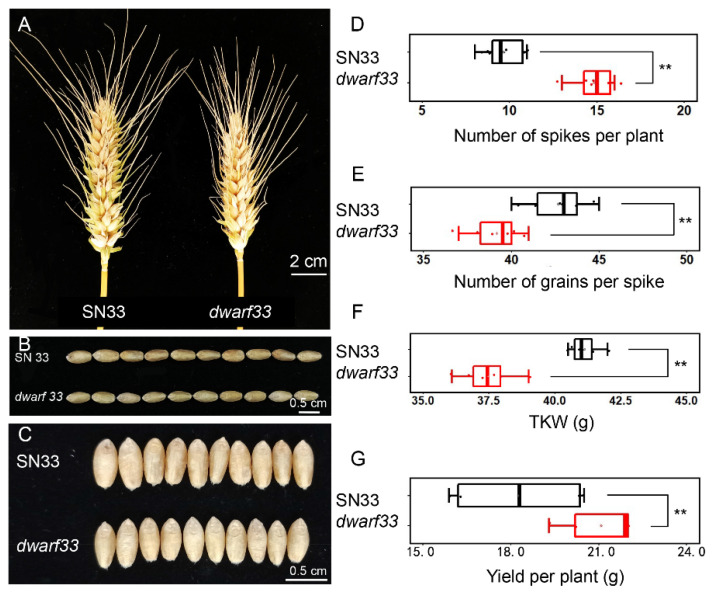
Agronomic trait analysis of the mutant *dwarf33* and wild-type SN33. (**A**) The spike of SN33 and *dwarf33*. (**B**,**C**) The grain characteristic of SN33 and *dwarf33*. (**D**) The number of spikes per plant of SN33 and *dwarf33*. (**E**) The number of grains per plant of SN33 and *dwarf33*. (**F**) The TKW of SN33 and *dwarf33*. (**G**) The yield per plant of SN33 and *dwarf33*. Asterisks “**” indicate significant difference at *p* ≤ 0.01.

The wheat mutant *dwarf33*, identified from an NaN_3_-mutagenized library in an SN33 background, exhibited shorter plant height compared to wild-type SN33 from the jointing stage (Figure 1A–C). At the grain filling stage, *dwarf33* was 63.3 cm tall, which was 22.4% shorter than SN33, while there was not significant difference in the number of internodes and spike length between them (Figure 1D–F). Compared to SN33, internode lengths of *dwarf33* were shorter by varying degrees, especially the second and fifth internodes, which decreased by 6.8 cm (30.3%) and 2.2 cm (40.9%), respectively (Figure 1F). As shown in Figure 2, there was no significant difference in spike length between *dwarf33* and SN33 (Figure 1F and Figure 2A). The grain length and width of *dwarf33* was close to that of SN33 (Table S3). The grain number per spike and TKW were lower than in SN33, but the spike number per plant was greater than in SN33 (Figure 2D–F). In addition, the results indicate that *dwarf33* was more sensitive to irrigation than SN33. The plant height of *dwarf33* decreased by 17.2 cm (27.2%) in a drought environment compared to that in an irrigated environment. However, the SN33 decreased by only 14.9 cm (18.3%) (Table 1). 

### 2.2. Cytological Observation of Internode Cells

**Figure 3:**
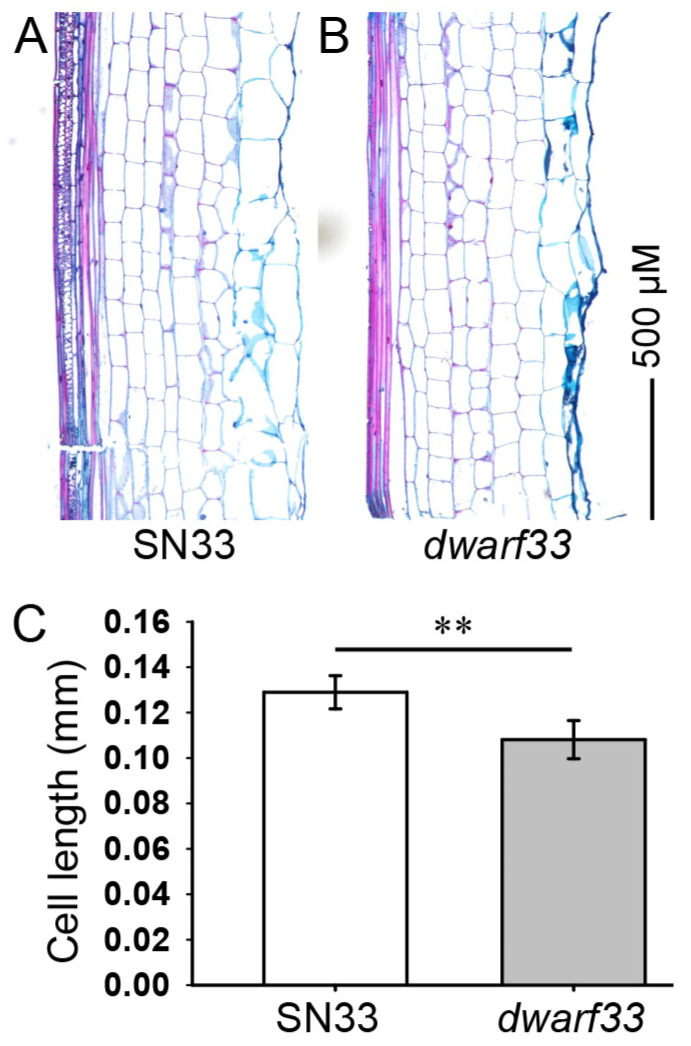
Internode cell length of SN33 and *dwarf33*. Asterisks “**” indicate significant differences at *p* ≤ 0.01.

As shown in Figure 1D,F, the second internode under the spike exhibited the largest difference in length between *dwarf33* and SN33. Therefore, the cell length of the second internode was measured to reveal the differences in cell characteristics between *dwarf33* and SN33. The results indicate that the cell length of the mutant *dwarf33* was significantly less than that of SN33. The cell length decrease in *dwarf33* reached approximately 15% (Figure 3C).

### 2.3. Coleoptile Length Measurement

**Figure 4:**
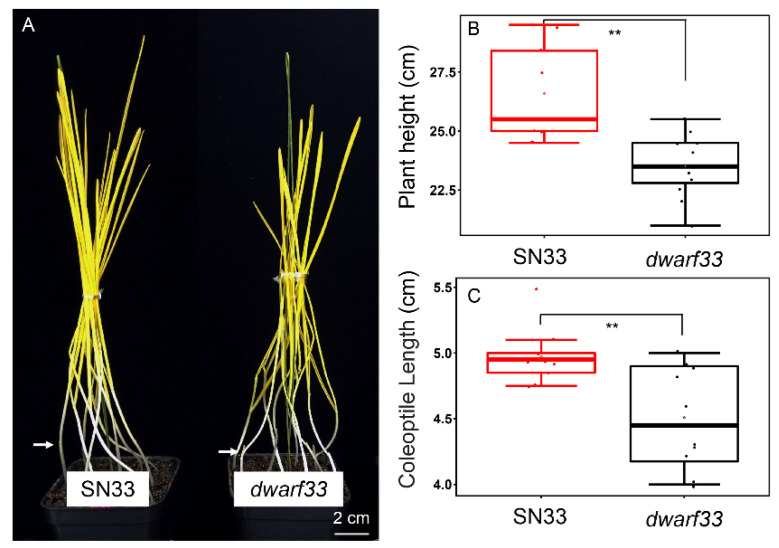
Coleoptile length and plant height in the dark environment. (**A**,**C**) Coleoptile length of SN33 and *dwarf33*. (**B**) Plant height of SN33 and *dwarf33*. Asterisks “**” indicate significant differences at *p* ≤ 0.01.

Coleoptile length is an important trait because it is closely associated with seedling vigor. Therefore, the coleoptile length of *dwarf33* and SN33 was measured under dark conditions. As shown in Figure 4A,C, the average length of coleoptiles in *dwarf33* was 4.5 cm, whereas the coleoptile length of SN33 was 4.9 cm. The coleoptile length of *dwarf33* decreased by 9.3% compared to SN33.

### 2.4. Dwarf Phenotype of dwarf33 Is Controlled by a Recessive Gene

For the genetic assay, the F_2_ population of a cross between *dwarf33*/SN33 and SN33/*dwarf33* were generated and analyzed. The phenotype of all the F_1_ plants of the reciprocal cross between *dwarf33* and SN33 resembled the wild-type SN33. This result revealed that the dwarf phenotype of *dwarf33* was recessive compared to the tall-plant phenotype. In the F_2_ population of *dwarf33*/SN33, the number of dwarf plants, similar to that of *dwarf33*, was 125 plants. The number of tall plants, similar to SN33, was 435 plants. The segregation ratio of the dwarf plants and tall plants in the *dwarf33*/SN33 population was 1:3. The dwarf plants and tall plants in population SN33/*dwarf33* displayed a 3:1 segregation as well (Table 2). Overall, the genetic analysis suggested that the dwarfism trait in *dwarf33* was controlled by a pair of recessive genes, which was named *Rht-SN33d*.

### 2.5. Dwarf Gene Mapping Based on BSA and the Wheat SNP Chip

**Figure 5:**
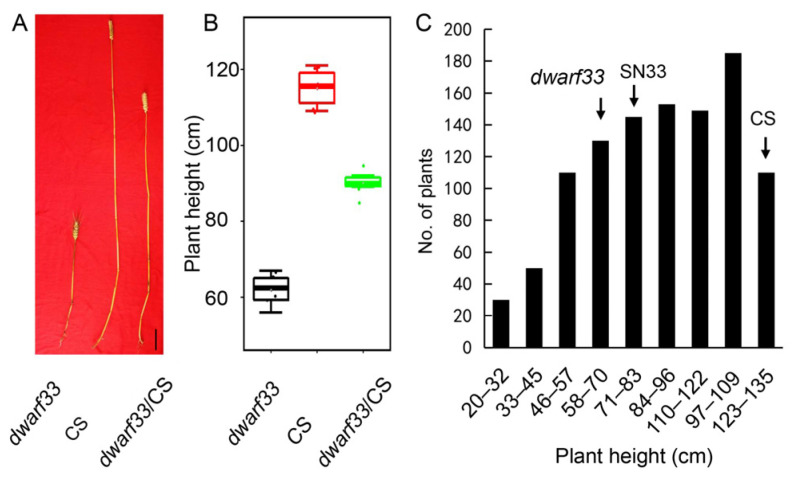
Plant height of the *dwarf33*/CS F_2_ population. (**A**) Plant height of *dwarf33*, CS and F_1_ of cross *dwarf33*/CS. (**B**) Plant height distribution of dwarf33, CS and F_1_ of cross *dwarf33*/CS. (**C**) Plant height distribution of F_2_ of cross *dwarf33*/CS. Label = 10 cm.

**Figure 6:**
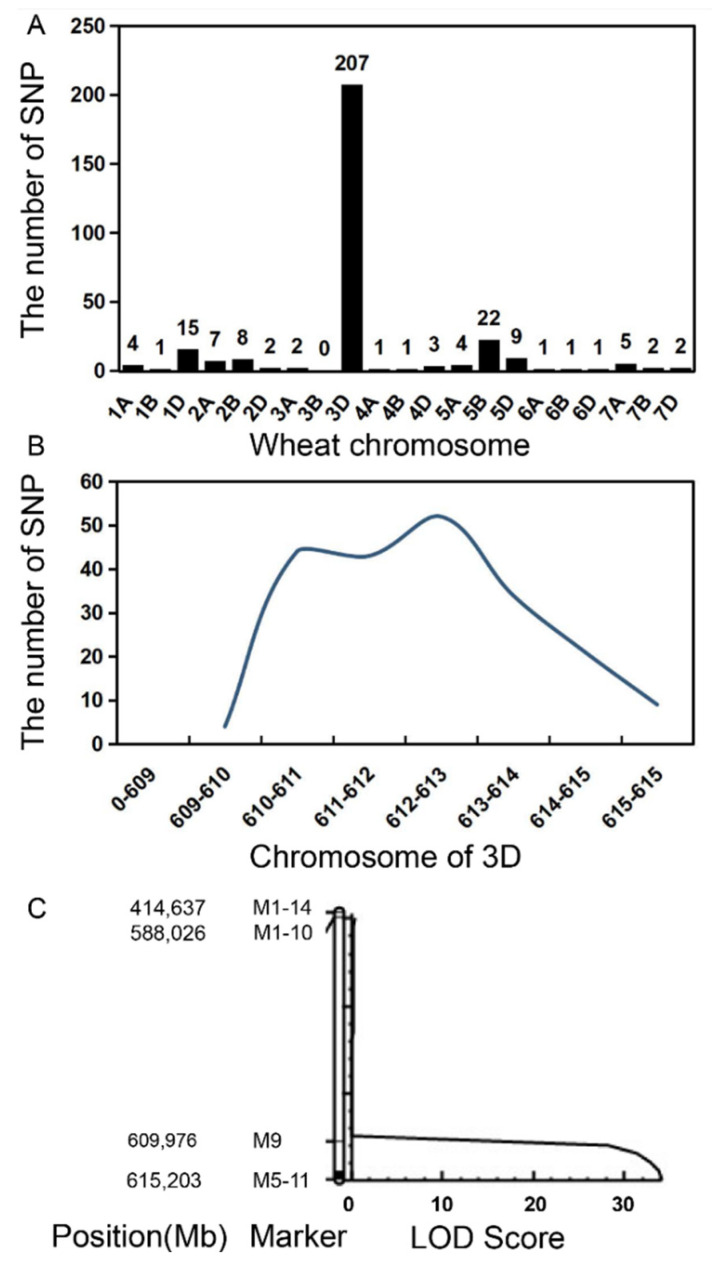
SNP distribution and QTL mapping. (**A**) The SNPs between dp and hp at wheat chromosome. (**B**) Numbers of SNPs per 10 Mb in chromosome 3D. (**C**) LOD value of QTL.

*Rht-SN33d* was mapped based on the F_2_ population derived from the *dwarf33*/CS cross. In the *dwarf33*/CS cross, the plant height of F_1_ was 89 cm on average, which was significantly higher than *dwarf33* and lower than CS. Plants with a height lower than SN33 were defined as dwarf plants, and those with a height taller than SN33 were defined as wild-type plants (Figure 5). A total of 19,416 homozygous SNPs were identified between the parents *dwarf33* and CS. Only 287 of the 19,416 SNPs were identified between dp and hp, and 207 of the 287 SNPs between dp and hp were located at 609.9–615.2 Mb of wheat chromosome 3D (Figure 6A,B). KASP markers were designed to create a genetic map, as shown in Figure 6C. A QTL with a LOD value of 34 was detected. Because of the low density of SNPs from the wheat 55K SNP chip, few SNPs were identified and successfully converted to KASP markers, especially the near the end of the chromosome of 3D. Hence, wheat exome capture sequencing was used to identify SNPs between *dwarf33* and CS to design more KASP markers. Only the plants lower than *dwarf33* could be determined to contain an *Rht-SN33d* homozygous genotype in the F_2_ population. For fine mapping of the gene *Rht-SN33d*, only the plants lower than *dwarf33* were used to detect the genetic recombination. Finally, the mutant gene was limited to an ~1.3 Mb interval (612.3–613.6 Mb, Tables S1 and S2) at the end of the wheat 3D chromosome.

In addition, the reported dwarf gene *RhtD1b* was detected in SN33, which is widely used in commercial varieties. To compare the dwarfing effect between *Rht-SN33d* and *RhtD1b*, the KASP marker “*KASP-Rht2*” was used to identify the genotype of the *dwarf33*/CS F_2_ population [18]. As shown in Table 3, *Rht-SN33d* presented different dwarfing effects in different backgrounds of *Rht2*. The average plant height, with genotype *Rht-SN33d*, decreased by 36.0 cm (30.2%) compared to the plants with genotype *Rht-SN33d* under the background *RhtD1a* in the *dwarf33*/CS F_2_ population. However, it decreased by 31.7 cm (29.2%) and 26.4 cm (30.6%) under the *RhtD1a*/*RhtD1b* and *RhtD1b* backgrounds, respectively, in the *dwarf33*/CS F_2_ population. Furthermore, the dwarfing effect of *Rht2* was analyzed in different genotypes of *Rht-SN33d* in the *dwarf33*/CS F_2_ population. In the background of *Rht-SN33*, *Rht-SN33d*/*rht-SN33d* and *rht-SN33d*, the average plant height of plants with *RhtD1b* decreased by 33.2 cm (27.8%), 29.0 cm (26.1%) and 23.6 cm (28.3%), respectively.

### 2.6. Candidate Gene Prediction

The region that contained the candidate gene was limited to a ~1.3 Mb interval (612.3–613.6 Mb) on the 3D chromosome. Twenty-two high-confidence genes were predicted in the candidate region based on the Chinese Spring wheat genome (Table 4). Interestingly, a GA biosynthesis related gene *TraesCS3D02G542800* was found that encoded gibberellin 2-beta-dioxygenase. GA and related metabolic intermediates are widely involved in plant height regulation. Therefore, *TraesCS3D02G542800* was predicted as the candidate gene. Thereafter, we cloned the CDS of *TraesCS3D02G542800* from *dwarf33* and SN33. The result indicated that there was no difference in the CDS of *TraesCS3D02G542800* between *dwarf33* and SN33 (detail sequence was listed in Supplementary materials).

### 2.7. Expression Pattern of Rht-SN33d

**Figure 7:**
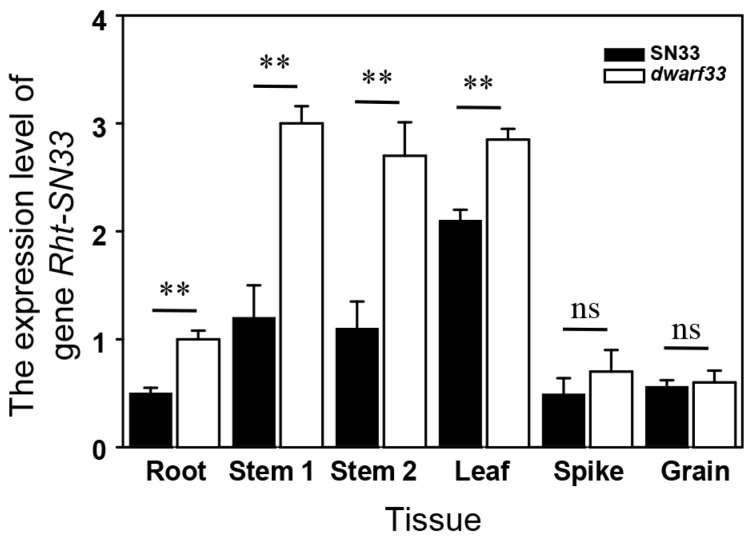
The expression pattern of the *Rht-SN33d* gene in SN33 and *dwarf33*. Stem 1: the stem was collected at the jointing stage; Stem 2: the stem was collected at heading stage. “ns” indicates no significant differences; Asterisks “**” indicate significant differences at *p* ≤ 0.01.

There were another two homoeologous genes of *Rht-SN33d* on chromosomes 3A and 3B that exhibited highly conserved DNA sequences. A sequence-specific primer for qPCR could not be designed to distinguish their expression. Therefore, the expression result from the primer pair Rht-SN33-F/R represented the total expression level of *Rht-SN33* on chromosomes 3A, 3B, and 3D. The results indicate that the expression pattern of *Rht-SN33* was tissue-specific (Figure 7 and Figure S1), and the stem and leaf exhibited higher expression levels than the root, spike, and grain. Furthermore, the expression level in *dwarf33* was significantly higher than that in SN33 for the roots, stems and leaves. 

## 3. Discussion

Dwarfism genes can reduce plant height, enhance lodging resistance, and increase tolerance to higher water and fertilizer which have been widely used to improve wheat agronomic traits and increase wheat yield. The first “Green Revolution” introduced dwarf genes into cultivars, which improved lodging resistance and yield [2].

At present, *Rht1* and *Rht2* [3] and *Rht8* and *Rht24* are widely applied in commercial varieties. It should be noted that the few dwarfism genes that are widely used could easily lead to the loss of genetic diversity and introduce adverse effects of dwarf genes into commercial varieties [19]. Better water and fertilizer conditions are indispensable condition for obtaining higher wheat yields [17]. Previous studies indicated that *Rht-B1b* and *Rht-D1b* could reduce plant height by ~23% [20]. Another dwarf gene, *Rht8*, effectively reduced plant height by approximately 20% [7]. In this study, the plant height of mutant *dwarf33* decreased by ~22.4% compared to wild-type SN33 (Figure 1E). Therefore, the dwarf gene in *dwarf33* showed a similar dwarfing effect on plant height to that of *Rht1* and *Rht2*, but slightly larger than that of *Rht8*. Interestingly, although *Rht-SN33d* has a similar dwarfing ability to *Rht2*, it only reduced coleoptile length by 9.3% (Figure 4C). *Rht1* and *Rht2* not only reduced the plant height but also shortened the coleoptile by approximately 20–30% [21,22]. A previous study indicated that some dwarf genes not only generate short plant height but also decrease the grain number per spike [23]. Spike length showed no significant difference between *dwarf33* and SN33 (Figure 1F). Although, the grain number per spike of *dwarf33* was less than SN33, the spike number per plant was more than SN33 (Figure 2D). These results suggested that *dwarf33* contained a dwarf gene that had a smaller negative effect on coleoptile length and spike development. *dwarf33* is a potential resource for seedling vigor improvement in wheat dwarfing breeding. 

The plant hormone GA is important in plant height regulation. In GA-mediated plant height regulation, it can form a complex with GID1 and DELLA, while SCF can degrade DELLA by this complex through ubiquitination protein degradation, thereby relieving growth inhibition by DELLA. However, mutant DELLA cannot be degraded, therefore inhibiting growth [11,12]. In this study, the candidate gene was in the ~1.3 Mb region on the long arm of chromosome 3D. Gene prediction showed that this region contains a gene *TraesCS3D02G542800* which encodes Gibberellin 2-beta-dioxygenase. Previous studies indicated that Gibberellin 2-beta-dioxygenases can catalyze the deactivation of bioactive GAs or their precursors [24]. Therefore, changing the GA content by modifying GA2ox genes is a potential tool for plant type improvement. For example, overexpression of GA2oxs could decrease liliaceous monocotyledon Tricyrtis sp. plant height [25]. The dwarfing phenotype was observed in the GA2ox genes PvGA2ox5 and PvGA2ox9 overexpression switchgrass lines, which were considered for plant architecture improvement [26]. In wheat, *Rht24* encodes GA2ox-A9 which is widely used for plant modification [6]. These results suggest that *TraesCS3D02G542800* may be the candidate gene of *Rht-SN33d*. 

As mentioned above, *Rht-SN33d* was mapped at the end of the 3D chromosome. Previous studies revealed that dwarfing genes are distributed in multiple wheat chromosomes. *Rht1* is located in 4B, *Rht2* in 4B [3], *Rht4* in 2BL, *Rht5* in 3BS, *Rht12* in 5AL, *Rht13* in 2DS [5], *Rht24* in 6A [6], and *Rht8* in 2DS [7,8] and so on. In our study, *Rht-SN33d* was located in the end of 3DL and is indicated as a new dwarf gene. However, there was no difference in the CDS of *TraesCS3D02G542800* between *dwarf33* and SN33. Previous studies have shown that structural variation may alter local chromatin states and, in turn, affect gene expression. For example, the deletion of a large fragment of approximately 304 kb near the *TaMLO-B1* site changed chromatin states and upregulated the expression level of the upstream gene *TaTMT3* [27]. Interestingly, the expression level of *Rht-SN33* in *dwarf33* was significantly higher than that in SN33 in the stem at the jointing stage and heading stage (Figure 7). As noted before, 2-beta-dioxygenases can inactivate GAs [24]. This suggests that sequence variation or methylation modification may have occurred in the promoter, and another possibility is that the expression pattern of *TraesCS3D02G542800* was affected by the mutation of nearby sites. Further experiments should verify these predictions.

In conclusion, the dwarf wheat *dwarf33* was identified in the SN33 mutant library. The *Rht-SN33d* gene was mapped in 3DL, the results indicating that this is a new locus involved in plant height regulation. *Rht-SN33d* has a similar dwarfing effect on plant height reduction to *Rht1* and *Rht2* but has a weaker negative effect on coleoptile elongation. Furthermore, *TraesCS3D02G542800* was predicted as the possible candidate gene encoding a gibberellin 2-beta-dioxygenase. Although the molecular mechanism of *Rht-SN33d* in *dwarf33* remains to be further studied, it is expected to provide new material for high yield and lodging resistance breeding. 

## 4. Materials and Methods

### 4.1. Plant Material

The dwarf mutant *dwarf33* was identified from the Shaannong33 (SN33) mutant library induced by 10 mM NaN_3_ (80115560, sinopharm, Shanghai, China) following the method of [28]. Wild-type SN33 is a superior commercial wheat variety that is widely cultivated in Shaanxi province and partly in the Yellow and Huai River Valleys of China. An F_2_ population with 1030 plants derived from *dwarf33*/CS (Chinese Spring wheat) was used to map the mutant gene, which was named *Rht-SN33d*. Parents and F_2_ individuals were planted in an experimental field in Northwest A&F University, Yangling, Shaanxi province in the year 2019 to 2020. The rows were 2 m long and 0.25 m apart, with 20 seeds per row. Normal irrigation involved irrigating once before and once after winter. Drought was based on irrigating by natural rainfall.

Leaf tissue was collected and stored at −20 °C for genomic DNA extraction. At the jointing stage (marked Stem 1) and heading stage (marked Stem 2), the second internode below the spike was collected and stored at −80 °C for RNA extraction, and some internodes were stored in FAA (formalin acetic acid ethanol) solution at 4 °C for paraffin embedding.

### 4.2. Phenotypic Assessment

At the filling stage, plant height is stable. The plant height of parents and F_2_ individuals was measured (2020 Yangling). Plant height was considered to be the distance from the ground to the top but did not include the length of the awn. Seedling height was defined as the distance between the seed to the top of the seedling. The data on plant height, internode length, and seed characteristics (such as seed length, width, and TKW), were collected from six independent plants (2019 Yangling). 

For coleoptile length measurements, the seed was germinated in water, then germinating seeds were planted in 7 × 7 × 7.3 cm (length × width × height) flowerpots, and cultivated under dark conditions at 25 °C. After 14 days l, the length between the seed and the top of the coleoptile was measured.

### 4.3. DNA and RNA Isolation and RT-PCR and RT-qPCR

DNA was isolated from wheat leaves following the CTAB method [29]. Total RNA was extracted using RNAiso Plus reagent (Takara Biomedical Technology, Beijing, China) according to the manufacturer’s instructions. Reverse transcription was performed using EasyScript^®^ One-Step gDNA Removal and cDNA Synthesis SuperMix (Transgen, Beijing, China). RT-qPCR was performed using TransStart^®^ Green qPCR SuperMix (Transgen, Beijing, China). RT-qPCR was performed using primers for *Rht-SN33*, where the *TaGAPDH* gene was used as an internal control (Table S1). Amplification was performed with one cycle of 2° min at 94 °C and 40 cycles of 10° s at 94 °C, 30° s at 60 °C and 1° min at 72 °C using a CFX96 Real Time PCR System (Bio-Rad, Hercules, CA, USA). Kompetitive allele-specific PCR (KASP) assays were performed following standard KASP guidelines (LGC Genomics, Middlesex, UK). All PCRs for cloning or sequencing were performed using a 2× Phanta^®^ Flash Master Mix (Vazyme, Nanjing, China). The data of gene expression were collected from three independent plants, the data from each plant representing three technical repetitions.

### 4.4. Microscopic Observation of Stem Tissue

Paraffin embedding of samples was performed using the method described by Weigel and Glazebrook [30]. Observation was performed on a Nikon Eclipse E100 microscope (Nikon Co., Ltd., Tokyo, Japan). Cell size was measured from three independent plants of *dwarf33* and SN33.

### 4.5. Marker Design and Gene Mapping

First, a dwarf DNA pool (dp) was created from 50 dwarf plants of the F_2_ population of *dwarf33*/CS in which the plant height was lower than that of *dwarf33*. A high-DNA pool (hp) was created from 50 dwarf plants from the same population in which the plant height was taller than *dwarf33*. Subsequently, single nucleotide polymorphisms (SNPs) were identified using a wheat 55 K SNP array from CapitalBio Technology (Beijing, China). The protocol for KASP marker design was based on the method developed in a previous report (Ramirez-Gonzalez et al. 2015). Then, QTLs and LOD values were detected by QTL IciMapping V4.1 [31] based on 1030 F_2_ plants derived from the cross between *dwarf33* and CS. Because of the low SNP density of the wheat 55K SNP chip, to identify more SNPs between *dwarf33* and CS, Exome capture sequencing was used to call the SNPs based on the sequence of Chinese Spring 1.1 [32,33]. Lastly, only the plants which were shorter than *dwarf33* were used to fine mapping the gene *Rht-SN33d*.

### 4.6. Gene Prediction and Cloning

Based on gene mapping, the candidate gene was limited to a certain chromosome region. The high-confidence genes were predicted based on the genome annotation of Chinese Spring v1.0 (WheatOmics 1.0, http://202.194.139.32/jbrowse.html, accessed on 5 May 2019) [34]. The primer pair Rht-SN33d-F1/R1 was used to isolate the CDS sequence of the candidate gene using the 2× Phanta Flash Master Mix (Vazyme, Beijing, China). The reaction system involved: step 1: 98 °C, 90 s; step 2: 98 °C, 10 s; step 3: 60 °C, 15 s; step 4: 72 °C, 15 s; 32 cycles from step 2 to step 4 and then maintenance at 12 °C. Thereafter, the PCR product was cloned into the vector using a Universal Ligation Mix kit (Vazyme, Beijing, China) following the manual. The DH5α spot selected on an LB media (50 mg·L^−1^ ampicillin) plate was used to identify the sequence of the candidate gene.

### 4.7. Statistical Analyses

The results are shown as mean ± standard deviation (SD) from at least three replicates (the details of replicates are listed in the relative section). A two-tailed Student’s *t*-test was used to analyze the significance between samples by using SPSS. *p*-value < 0.05 (*) and *p*-value < 0.01 (**) are regarded as significant.

## Figures and Tables

**Table 1:** Plant height of SN33 and *dwarf33* under different irrigation conditions.

Name/Plant Condition	SN33 (cm) ± SD	*dwarf33* (cm) ± SD	*p*-Value
Normal irrigation	81.6 ± 2.3	63.3 ± 3.1	5.4 × 10^−11^
Drought	66.7 ± 4.3	46.1 ± 2.7	3.6 × 10^−6^
Plant height decrease (%)	18.3	27.2	

SD: standard deviation; Plant height decrease (%) was calculated by plant height under normal irrigation minus plant height under drought)/plant height under normal irrigation.

**Table 2:** Phenotypic analysis of the populations *dwarf33*/SN33 and SN33/*dwarf33*.

Population	Generation	No. Plants	No. Plants		Expected Ratio	χ^2^	*p*-Value
			d	T			
*dwarf33*	P1	10	10	0			
SN33	P2	10	0	10			
*dwarf33*/SN33	F1	31	0	31			
	F2		125	435	1:3	1.11	0.708
SN33/*dwarf33*	F1	30		30			
	F2		75	262	1:3	0.67	0.586

*p*: parent; T: tall plant; d: dwarf plant. The data were collected from Yangling, Shaanxi, 2020.

**Table 3:** Phenotypic analysis of the F_2_ population of *dwarf33*/CS.

Genotype	*RhtD1a*	*RhtD1a/1b*	*RhtD1b*	(*RhtD1a*) − (*RhtD1b*)
*rht-SN33d*	119.4	108.6	86.2	33.2
*Rht-SN33d*/*rht-SN33d*	111.1	106.2	82.1	29.0
*Rht-SN33d*	83.4	76.9	59.8	23.6
(*rht-SN33d*) *−* (*Rht-SN33d*)	36.0	31.7	26.4	

Note: Data were from 2020, Yangling, Shaanxi Province; unit of plant height: cm; “−”: minus.

**Table 4:** Highly confident genes in 612.3–613.6 Mb of wheat 3D chromosome.

Chr	Start	Stop	ID	Description
chr3D	612311199	612312868	TraesCS3D02G541100	TOX high mobility group box protein
chr3D	612312442	612318234	TraesCS3D02G541200	Vacuolar fusion protein MON1
chr3D	612604471	612605205	TraesCS3D02G541300	Enoyl-[acyl-carrier-protein] reductase
chr3D	612763464	612764795	TraesCS3D02G541400	Regulator of chromosome condensation (RCC1) family with FYVE zinc finger domain-containing protein
chr3D	612859406	612859915	TraesCS3D02G541500	Disease resistance protein RPP13
chr3D	612878354	612881039	TraesCS3D02G541600	Disease resistance protein (NBS-LRR class) family
chr3D	612886487	612898293	TraesCS3D02G541700	ABC transporter B family protein
chr3D	612899761	612906345	TraesCS3D02G541800	Chaperone protein
chr3D	613057363	613058851	TraesCS3D02G541900	Plant/T31B5-30 protein
chr3D	613076025	613076727	TraesCS3D02G542000	Pectinesterase inhibitor
chr3D	613119248	613126869	TraesCS3D02G542100	SNARE-interacting protein KEULE
chr3D	613357955	613358990	TraesCS3D02G542200	Extra-large guanine nucleotide binding family protein
chr3D	613383728	613390403	TraesCS3D02G542300	Protein kinase-like protein
chr3D	613480307	613481622	TraesCS3D02G542400	lectin-receptor kinase
chr3D	613569028	613570372	TraesCS3D02G542500	Disease resistance protein (TIR-NBS-LRR class) family
chr3D	613588995	613592124	TraesCS3D02G542600	Plant cadmium resistance protein
chr3D	613594052	613594433	TraesCS3D02G542700	Dirigent protein
chr3D	613597346	613599060	TraesCS3D02G542800	Gibberellin 2-beta-dioxygenase
chr3D	613601793	613602401	TraesCS3D02G542900	Dirigent protein
chr3D	613607577	613610693	TraesCS3D02G543000	transmembrane protein
chr3D	613618086	613622258	TraesCS3D02G543100	Chaperone protein
chr3D	613631318	613633402	TraesCS3D02G543200	SKP1-like protein

## Data Availability

The data presented in this study are available on request from the corresponding author.

## Supplementary Materials

The following supporting information can be downloaded at https://www.mdpi.com/article/10.3390/ijms24010583/s1.
