# Supplementary material for: Agronomic Trait Analysis and Genetic Mapping of a New Wheat Semidwarf Gene Rht-SN33d
Source: Int J Mol Sci. 2022 Dec 29;24(1):583. doi: 10.3390/ijms24010583 (PMC9820066; doi:10.3390/ijms24010583)
Supplement: Supplementary file 1 [file ijms-24-00583-s001.zip › Supplemental materials.pdf]

The sequence of TraesCS3D01G542800 (*Rht-SN33d*) in cs, dwarf33 and SN33.

TCAGTTGTAGGTGTCCACTTTATTATATAAACAAAAGAGTAAAGGTTGTCCCTTTGCCTGCTCTGCTCTCCTCTGTTGTATCTAGATCCA

TCAACAGGACTGCCGCGTGAATCGTGATGCAGCAGCAGGAGATCCATCTTCGTCGATTCATGGAGCATCAGCCAGACGAGTGCGCGC

TGGAGGAGCTGGAGCTGCCCACTGTAGACCTCGAGGCGGAGGAGCCGAGCCTGACGGAGCAGCTGGCGGCGGCGTGCCGGGACCC

GGGCGTGTTCCGGCTGGTCAACCACGGCGTCCCCTGCGACCTCACCGCCCGCCTGTTCGGGCTGGCGCGCGGGCTCCTTGAGCTGGA

CGCGGCCAACAAGTCCCGGCTGCCCGGCTACTTCTGTGGCACGCCGGCGCTGGCGGCGCTCCCTGTCAAACAGCTCAATTGGCTCGA

GGGGCTGCACGTCGAGGCCGACGACACCGGCGACCGTTCTCATTCTTCTGCTGACGCTGCTGACGGTGAAGCAGGCGGCAGTGCCT

TGGCGGAGTTCATGGAGGCGGTGAGCGGGGAGTACGTGGCGCACATGGCGCGCATCGCTCGCAAGCTGTTCGACACCCTGGCCTGC

GGCGAGCTGGGCCTGGACGAGGAGCAGCGGGCGTCGTACCTGACGGAGCGCGGCTGCATCTTCCGCGCGTACCGGTACCCGGCTAC

TGCGTCGGGCGCGGGGCGGCGGCAGCTGGGGATGGAGGCGCACACGGACAGCTCGGTGCTGTCGATCCTGAACCAGGACAGGGTG

GGCGGCCTGCAGGTGCTCTATGGTGGTAGGTGGCTTGCGATGCGGCCTATGGAGGGCGCGCTGGTGGTGAACGTGGGCGACATGCTG

CAGGCGATGAGCGGGGGCGCGTACCGGAGCCCGGAGCACCGGGTGGTGGCGCCGGGCTGGACGGAGGTGGGCAGGATGTCGCTCT

GCTACTTCGCGTTCCCGCAGGAGGACGCCGTCATCGTCGGCCCGCCGTCAGCTTGTCGTCAGGAGGAGTTGTACAGGGCGTTCAGCT

ACCGTGAGTTCCGGGAGCAGGTGCAGGCGGACGTGAAGGCCAGCGGCTCCAAGGTCGGCCTCGCCCGATTCCGCGTCCCCGTGAGT

CAATCATAGTCATGTTCATTGGACGGGACGGACGGCGCCTTGGAACAAGCCTGATCGATCCATTCTTCT

CDS was marked by gray; forward primer and reverse primer for TraesCS3D01G542800 full length amplification were marked red and green respectively.

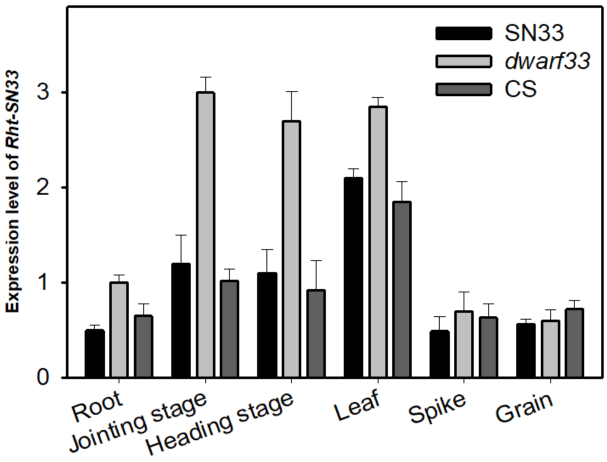

Figure S1. The expression level of Rht-SN33 in SN33, mutant dwarf33 and CS at different tissues.
